# An Explanation of the Signs and Symptoms of Pregnancy, with Some Other Papers Connected with Midwifery

**Published:** 1857-07

**Authors:** 


### BOOK NOTICES.

*An Explanation of the Signs and Symptoms of Pregnancy, with some other Papers connected with Midwifery.* By W. F. Montgomery, A. M., M. D., etc., etc. From the second London edition; published by Blanchard & Lea, Philadelphia. For sale by Keen & Lee, Chicago.

The work now before us is a model of style and elegance, and shows the good taste of its publishers in an eminent degree.

The typographical appearance of this reprint speaks much in favor of the great change which has taken place in the getting up of medical books within the last few years. The colored plates representing the corpus luteum exhibit an accuracy and finish highly commendable to the lithographic establishment of Mr. T. Sinclair. The work is a good sized volume, containing over 500 pages, exclusive of preface and index, which are divided into thirteen chapters; each chapter comprising the consideration of several topics, subdivided and arranged in regular order.

Although a second edition, the present treatise may almost, as our author says, be considered a new book. Indeed, enough has accumulated since 1837, the date of the former edition, to render it necessary, in order to present a complete work, which should represent our actual knowledge on this subject, to make many new additions and modifications.

Chap. 1. "General Observations on the State of the Female System during Pregnancy." This chapter is given preparatory to entering more fully into the detail of particular signs, that we may the better understand and appreciate their true significance and import. The changes produced in the female system during pregnancy, are well known to give rise to various mental and physical phenomena, which require no great effort to be comprehended and explained on well established physiological and pathological principles. That part of the chapter which treats of the increased susceptibility of the nervous system and mind of the pregnant woman, especially as regards impressions made by external objects, and the influence thus exercised on the physical organization and mental constitution of the child, and also, the relation existing between pregnancy and disease, is discussed with more than usual interest and benefit. Dr. M. does not hesitate to deny the foolish absurdity of the popular doctrine that effects are produced on the child by the mother's imagination, or to maintain his belief that powerful impressions on the mother's mind or nervous system may injuriously affect the fœtus still lodged in its mother's womb. To illustrate by example the effect of this mental influence on the child, he has given the details of several cases, two of which occurred under his own observation, and are of so much interest, that we beg to rehearse them.

Case 1. A lady, pregnant for the first time, to whom was recommended frequent exercise in the open air, declined going out as often as was thought necessary, assigning as her reason that she was afraid of seeing a man whose appearance had greatly shocked and disgusted her; he used to crawl along the highway on his hands and knees, with his feet turned up behind him. Dr. M. afterward attended this lady in her confinement, and her child, which was born a month before its time, and lived but a few minutes, although in every other respect perfect, had its feet malformed and defective in the same way as those of the cripple who had alarmed her.

Case 2. Mrs. A., the wife of a clergyman, came to town for her confinement, being very uneasy in her mind from an apprehension that her child would be born with a deformed hand. Her anxiety was induced by her having seen, during her pregnancy, a child with one of its hands deformed in such a way that it affected her mind very strongly, and produced the impression that her yet unborn infant would be similarly affected. Very soon after her delivery she expressed an anxious wish to see her child, which was brought to her wrapped up in a flannel in the usual way. She instantly drew out the child's arm, and exclaimed, with a look and tone of horror, "Oh, the dreadful hand!" and there it certainly was, with exactly the same deformity as that which had excited her disgust and terror several months before. No explanation is given to account for these singular phenomena, though of their occurrence no doubt can be entertained. A more singular, and just as inexplicable a phenomenon is mentioned still further on in this same chapter. It is this:

Certain peculiarities of the male parent may be transmitted to the subsequent offspring of the same woman, begotten by another man; for instance, suppose a white woman to be first impregnated and have a child by a negro, and afterward have another child by a white man, it is more than probable that this second child will exhibit certain peculiarities belonging to the negro. In view of this fact Dr. M. asks, is it possible that a morbid taint, such as that of syphilis, for instance, having been once communicated to the system of the female, may long linger there, and influencing several ova, continue to manifest itself in the offspring of subsequent conceptions, when impregnation has been effected by a perfectly healthy man, and the system of the mother appearing to be at the same time and for a considerable period previously quite free from the disease? His opinion he gives in the affirmative. Two cases in point are given, one from Vidal, and one from Cazenave.

The remaining part of this chapter comprises the consideration of the relation between pregnancy and disease. It contains many important practical remarks on this subject, which deserve the careful consideration of the medical accoucheur. No one who has ever practised extensively but has often noticed the effect produced on all forms of disease occurring during gestation. The effect of pregnancy on phthisis has long attracted the attention of the profession, and called forth many an animated discussion in high places, but still remains unsettled. Dr. M. believes that those predisposed to phthisis, but who are not actually suffering, will be benefited by pregnancy, but on the other hand, where the disease already exists and has made some progress, injury will accrue from such a state.

Chap. 2. "Investigation of the Signs of Pregnancy—Legal and Social Relations—Different Sources of Evidences or Proofs of that State—Classification of Signs."

The importance of being able to decide on the existence or absence of pregnancy is among the first on the list of professional duties requiring a thorough knowledge and aptness in applying it. But if it is important, we must at the same time admit that it is difficult. When we read of such men as Corvisart and Baudelocque examining a case, and one pronouncing it encysted dropsy, and the other cancer uteri, and soon after the patient gives birth to a healthy child, we can easily conceive of difficulties only to be surmounted by a thorough understanding of the signs which indicate such a condition. Dr. M., though admitting this difficulty, believes mistakes arise far less from this acknowledged difficulty of the investigation than from the want of proper information, and the careless way in which examinations are conducted. He divides the symptoms into three classes—first, *presumptive;* second, *probable;* and third, *positive.* The certain or unequivocal symptoms are also three—first, active movements of the child unequivocally felt by the mother; second, its presence in utero ascertained by ballottement; and third, the pulsations of the fœtal heart. These signs, however, are unequivocal only in the affirmative, as their absence would not be proof positive of the non-existence of pregnancy.

Chapters 3 and 4. "Individual Signs." The first mentioned in detail is suppression of the menses, which, however, varies so much in health and disease as to be of little value. Several cases are given where pregnancy occurred before the appearance of the catamenia, and others where they were present only during that period. Nausea and vomiting, salivation, mammary sympathies, areola, and the secretion of milk, comprise the subjects discussed in the remainder of these two chapters. None of them need engage our particular attention at present, except the areola. The areola Dr. M. believes the most reliable of all the external symptoms, and, according to his experience, a state of pregnancy only can produce it. His description of a perfect areola is truly graphic, and much more minute and satisfactory than any we have previously seen. The most distinctive feature noticed by former writers has been the color, but on this Dr. M. places the least reliance. "The puffy turgescence, not alone of the nipple, but of the whole of the surrounding disk, and the development of the little granular follicles, with the developed state of the mammary veins, are the objects to which we should principally direct our attention." These chapters are closed by the consideration of "Mammary Sympathies," and the "Secretion of Milk," which are both taken as valuable presumptive, but not at all positive, proof of pregnancy.

Chap. 5. "Quickening and Motions of the Fœtus." This symptom, when present beyond all doubt, is one of the unequivocal signs of pregnancy, but there are many sources of error, and we should use every precaution before we give an opinion. The *period* of quickening, which so greatly engaged the attention of the profession at an early day, is yet indefinitely settled. Dr. M., who opens this chapter by saying that he reluctantly uses the word quickening, and only in compliance with a long established custom, believes the greatest number of instances will be found to occur between the end of the twelfth and sixteenth weeks after conception. He mentions one case where it occurred sixty-seven days after conception. Denman says, "Quickening happens at different periods of pregnancy, from the tenth to the twenty-fifth week, but most commonly about the sixteenth after conception." Hippocrates says that, "as a general rule the male fœtus is felt to move at three months, and the female at four." The period of quickening, then, being variable, the deception produced by other movements than that of a fœtus, renders it a symptom of no very great value.

Chap. 6. "Enlargement of the Abdomen and State of the Umbilicus."

Chap. 7. "Changes in the Uterus—State of the Os and Cervix Uteri—Size of the Uterus—Its Contents, Situation, and Consistence." These chapters comprise a complete review of the above subjects, and their relative importance as symptoms of pregnancy, with illustrations and the views of different authors, but seem to us to present no new features or points for comment, except it be Dr. M.'s statement, that though a corpus luteum *may* be found at each menstrual period, such an occurrence is *not necessary or invariable.* In this respect we believe Dr. M. differs from most writers on this subject. As regards the indications to be ascertained from the state of the cervix, Dr. M. considers them the most reliable, and least liable to error of any known to us. Besides their greater importance, they enable us to determine, in most instances, with considerable accuracy, the period of gestation.

Chap. 8. "Of the different modes of Examination, and the Method of Conducting them—Summary." This chapter is full of directions as to the best modes of conducting examinations, to ascertain the existence or absence of pregnancy, and is one of very great value. In regard to the auscultatory signs, after fully considering the time when they are most likely to appear, their situation, variation, and the most probable causes of deception, he concludes by confirming his previous conviction regarding their diagnostic value, by saying, that "without meaning to depreciate their great and acknowledged value, as an occasional means, he is still of the opinion that for those who are thoroughly conversant with the proper methods of examining doubtful cases, it will seldom be found necessary, if all the other more ordinary modes have been adopted with sufficient care." This opinion, however, is given with a reservation. Dr. Kluge, professor of midwifery at Berlin, and M. Jacquemin, of Paris, were the first to notice the dusky hue of the vagina as a symptom of pregnancy, which they declared to be *a sure test*. At the time Dr. M.'s previous edition was issued, he had directed so little attention to this sign that he did not speak decidedly in reference to it; since then, however, he has had ample opportunities of testing its value, and without hesitancy gives his opinion that "it is one of the most constant signs of pregnancy." This chapter closes with a brief review of all the symptoms, their time of appearance, the order in which they occur, and their relative value. Only two signs are given as proof positive of pregnancy; they are, "the pulsations of the fœtal heart," and "the movements of the child in utero." "In any case of a criminal or civil character, and involving property, reputation, or life, our decision ought to rest on no evidence that admits of doubt."

Chap. 9. "Examination of Substances expelled from the Uterus—Moles—Hydatids—The Membrane found in Dysmenorrhœa, and in other Conditions of Uterine Derangement—Membranous Formations from the Vagina." This chapter describes the process of examining substances expelled from the uterus, and the means of determining whether it be an ovum. Moles and hydatids Dr. M. believes do not occur except after sexual intercourse, and as a consequence of impregnation. With regard to the time that such formations may be retained in utero much discrepancy of opinion exists. Madame Boivin has tabulated thirty-two cases, making the average period between six and seven months. Dr. Ryan relates a case of hydatids which continued for fourteen years. Gardien, Desormeaux, and Velpeau admit that they may be retained many years, but Dr. M. believes the time limited from six to twelve months.

Chap. 10. "Accidental Circumstances—Beccaria's Test—State of the Blood, Urine, and Pulse." In the first part of this chapter is noticed certain peculiarities not unfrequently observable in pregnant women, and many curious instances are given. According to Beccaria there is a pecular kind of headache accompanying pregnancy, which he describes as an acute pulsating pain in the occipital region. Dr. M. has met with several cases where faintness or actual fainting occurred at certain times of the day. Some women, when pregnant, have a depraved appetite, or take a particular fancy to some form of diet, which, at other times, was particularly disagreeable; others take a fancy to particular objects and pursuits. One woman was invariably seized with an uncontrollable desire for building, another to eat ginger. These phenomena in first pregnancies are of little value, but in those who have born children, and suffered from them, they become valuable auxiliaries. This chapter is closed by considering the condition of the blood, urine, and pulse in pregnancy. The buffy coat of the blood, by many thought to be au invariable concomitant on pregnancy, Dr. M. regards as fallacious, and places the subject beyond all question. Kyestien, an element of the urine in certain cases of pregnancy, first described by M. Nauche, and afterward by M. Equisier, and by them thought to be an invariable product, and always present, Dr. M. disposes of in the same summary manner. Since 1831, the time M. Nauche first announced the discovery of this substance in the urine, it has received the attention of many eminent investigators down to the present day, and now, we believe, is pretty generally acknowledged to be not peculiar to pregnancy, but may occur under other circumstances, and even in men. No remarks on the pulse requiring notice.

Chap. 11. "Pregnancy under unusual Circumstances, of Age, Disease—Without Consciousness—Imperfect Intercourse—Secondary Ovum."

Chap. 12. "Spurious or Simulated Pregnancy."

Chap. 13. "Investigation after Death—Examination of the Uterus, and its Appendages—The Ovaries, Corpora Lutea, and Fallopian Tubes." These three chapters are full of interest and instruction, being a full survey of the subjects on which they treat, and many others of equal importance. The many circumstances connected with pregnancy in its relation to disease, and other conditions of the female economy, are well and ably commented on. A full description of the corpus luteum of pregnancy is given, as well as its distinguishing features from the corpus luteum of menstruation. We regret that our limits will not allow a more extended notice of these subjects, as we feel that they richly deserve and would well repay a careful review. The work is closed by three papers, written with much care, and comprehending a thorough investigation of the following subjects: First, "The Period of Human Gestation;" second, "The Signs of Delivery;" and third, "The Spontaneous Amputation of the Fœtal Limbs in Utero." Each of these subjects being of so great an importance, and of so much interest to the profession, have been fully discussed, and are made to appear plain and easy for observation and study. "The Period of Human Gestation," which has so long been the vexed and much disputed question, has, in our opinion, been so carefully studied by Dr. M. as to leave no longer room for discussion. He has proved by actual observation that labor may not only be premature but may be protracted. On both these questions he has given tables from different authorities, showing, beyond all doubt, that labor may be delayed one, two, or three weeks. So thoroughly is this subject considered, and so impartially, that we believe no one unprejudiced can read them without being, as we have been, fully convinced of their correctness. The last subject, "Spontaneous Amputation of the Fœtal Limbs in Utero," forms an important finish and excellent topic for closing a work which we have found so full of interest and instruction. The whole work, though rather large, is written in such a pleasing manner, and of so finished a style, as to be easily studied without much effort, and to certainly impart an amount of knowledge not to be obtained from any other source. We bespeak for it a generous reception and due appreciation by our professional brethren, who will find it a valuable addition to their libraries. G. K. A.

---

We have received from the publishers, Messrs. Blanchard & Lea, of Philadelphia, copies of Todd and Bowman's Physiology, and Churchill on Diseases of Women, by Condie; both valuable books, which we shall notice more at length hereafter.

We have also on our table several Monographs, Addresses, etc., which we have neither time nor space to notice in the present number.

